# Retrograde adenoviral vector targeting of nociresponsive pontospinal noradrenergic neurons in the rat in vivo

**DOI:** 10.1002/cne.21879

**Published:** 2009-01-10

**Authors:** Patrick W Howorth, Anja G Teschemacher, Anthony E Pickering

**Affiliations:** 1 Departments of Physiology and Pharmacology, University of BristolBristol, BS8 1TD, UK; 2 Department of Anaesthesia, Bristol Royal InfirmaryBristol, BS2 8HW, UK

**Keywords:** locus coeruleus, descending control, nociception

## Abstract

The spinal dorsal horn receives a dense innervation of noradrenaline-containing fibers that originate from pontine neurons in the A5, locus coeruleus (LC), and A7 cell groups. These pontospinal neurons are believed to constitute a component of the endogenous analgesic system. We used an adenoviral vector with a catecholaminergic-selective promoter (AVV-PRS) to retrogradely label the noradrenergic neurons projecting to the lumbar (L4–L5) dorsal horn with enhanced green fluorescent protein (EGFP) or monomeric red fluorescent protein (mRFP). Retrogradely labeled neurons (145 ± 12, n = 14) were found in A5-12%, LC-80% and A7-8% after injection of AVV-PRS-EGFP to the dorsal horn of L4–L5. These neurons were immunopositive for dopamine β-hydroxylase, indicating that they were catecholaminergic. Retrograde labeling was optimal 7 days after injection, persisted for over 4 weeks, and was dependent on viral vector titer. The spinal topography of the noradrenergic projection was examined using EGFP- and mRFP-expressing adenoviral vectors. Pontospinal neurons provide bilateral innervation of the cord and there was little overlap in the distribution of neurons projecting to the cervical and lumbar regions. The axonal arbor of the pontospinal neurons was visualized with GFP immunocytochemistry to show projections to the inferior olive, cerebellum, thalamus, and cortex but not to the hippocampus or caudate putamen. Formalin testing evoked c-fos expression in these pontospinal neurons, suggesting that they were nociresponsive (A5-21%, LC-16%, and A7-26%, n = 8). Thus, we have developed a viral vector-based strategy to selectively, retrogradely target the pontospinal noradrenergic neurons that are likely to be involved in the descending control of nociception.

The individual perception of pain may be dramatically influenced by the circumstances under which injury occurs (Wall, 1999). Thus, there are endogenous central neural mechanisms that regulate the transmission of nociceptive information according to the animal's behavioral state when an injury occurs. Since the initial report of electro-analgesia following brainstem stimulation (Reynolds, 1969), much attention has been focused on the mechanisms mediating the descending control of nociception (Stamford, 1995; Millan, 2002; Lumb, 2004). The pontine noradrenergic cell groups (A5, A6 [locus coeruleus, LC], and A7) are believed to form a key antinociceptive component of this system, as some of these neurons project to the dorsal horn of the spinal cord (Westlund et al., 1983) and release noradrenaline (NA) to suppress transmission of the pain message (Millan, 1997; Pertovaara, 2006).

Multiple strands of evidence support the role of the pontospinal noradrenergic neurons in mediating an antinociceptive action. Direct stimulation (electrical or chemical) of these NA nuclei (A5–7) in anesthetized animals produces antinociception (Jones and Gebhart, 1986a; Burnett and Gebhart, 1991; Yeomans et al., 1992). Alpha-adrenoceptor antagonists have been shown to block brainstem stimulation-evoked analgesia (Miller and Proudfit, 1990) and indeed to cause hyperalgesia under basal (unstimulated) conditions (Sagen and Proudfit, 1984). Similarly, ablation of the spinal NA projection with 6-OH dopamine has been reported to produce acute hyperalgesia (Fasmer et al., 1986). At a cellular level, extracellular recordings of dorsal horn nociceptive cells have shown LC stimulation to decrease noxious heat-evoked firing (Jones and Gebhart, 1986b). Intracellular recordings have shown that application of NA inhibits dorsal horn neurons by both pre- and postsynaptic actions at alpha2-adrenoceptors (North and Yoshimura, 1984; Sonohata et al., 2004). The intrathecal administration of alpha-adrenoceptor agonists is analgesic in animals (Reddy et al., 1980; Reddy and Yaksh, 1980) and humans (Eisenach et al., 1998). Furthermore, a number of clinically useful analgesics act by mimicking (clonidine/dexmedetomidine) or enhancing (amitriptyline/tramadol/duloxetine) the activity of the noradrenergic system.

This view of the pontospinal noradrenergic system has been questioned following some recent experiments using intrathecal administration of the toxin saporin conjugated to antidopamine β-hydroxylase (DBH) antibodies to ablate the NA neurons which did not demonstrate the expected pronounced hyperalgesia (Martin et al., 1999; Jasmin et al., 2003a). This may have been due to a number of factors, such as the loss of noradrenergic neurons with projections to other brain regions or compensatory responses to the loss of NA inputs. An alternative possibility could be that the ablation of a system capable of bidirectional modulation (Holden et al., 1999; Hedo and Lopez-Garcia, 2001; Millan, 2002) could produce a subtle net overall effect on nociception. Nonetheless, these findings have lead Jasmin et al. (2003b) to call into question the hypothesis that antidepressant analgesia (e.g., with amitriptyline) is mediated by an effect on the NA system.

The brainstem NA neurons have large axonal arbors with extensive collateralization that project to form a diffuse network of terminals throughout the entire central nervous system (CNS) (Dahlstrom and Fuxe, 1964; Palkovits and Brownstein, 1989). However, there is evidence of spatial specificity in the organization of the NA system (Foote et al., 1983); for example, the LC has a topographical organization and neurons exhibit specific target fields, e.g., spinal cord, cerebellum, hypothalamus, or cortex (Loughlin et al., 1986). Furthermore, the catecholaminergic innervation of the dorsal, ventral, and lateral horns of the rat spinal cord is reported to originate from the A7, LC, and A5 cell groups, respectively (Clark and Proudfit, 1991a,b;^1993^). This complex anatomical and functional organization may account for some of the differences seen between previous studies as a consequence of the difficulty of selectively activating or inactivating the pontospinal noradrenergic component in isolation.

In order to selectively target the pontospinal noradrenergic system, we have developed an adenoviral vector (AVV) strategy. This takes advantage of the ability to retrogradely transfect neurons using AVV and a catecholaminergic selective promoter (PRS) (Hwang et al., 2001; Lonergan et al., 2005) to drive expression of fluorescent proteins in pontospinal noradrenergic neurons. Using this technology, we have been able to define the anatomical organization of the pontospinal noradrenergic projection to the lumbar dorsal horn, which receives peripheral inputs from the hindpaw, and have shown that these neurons are activated following a persistent noxious stimulus.

## MATERIALS AND METHODS

Experiments were performed on 70 male Wistar rats (University of Bristol colony). All procedures conformed to the UK Animals (Scientific Procedures) Act 1986 and were approved by our institutional ethical review committee. Animals were group-housed with an enriched environment under a standard 12-hour light/dark cycle, with free access to food and water.

### Adenoviral vector construct and preparation

The AVV shuttle plasmids for AVV-PRS-EGFP and AVV-PRS-mRFP were constructed by inserting the 240-bp PRSx8 promoter sequence (Hwang et al., 2001) upstream of EGFP and mRFP sequences (Duale et al., 2005; Lonergan et al., 2005). AVVs were obtained by homologous recombination with helper plasmids and proliferation in HEK293 cells, followed by CsCl gradient purification, using standard techniques (Graham and Prevec, 1995). The AVV stocks were titered by an immunoreactivity “spot” assay (goat antihexon antiserum, Biodesign International, Kennebunk, ME) according to previously published protocols (Bewig and Schmidt, 2000; Duale et al., 2005) and titers were expressed in transducing units (TU) per mL.

### Surgical procedures

Wistar rats (130–200 g) were anesthetized (i.m. or i.p.) with ketamine (5 mg / 100 g, Vetalar, Pharmacia, Northamptonshire, UK) and medetomidine (30 μg / 100 g, Domitor, Pfizer, Kent, UK) until loss of hindpaw withdrawal reflex. The animal was placed in a stereotaxic frame and secured in atraumatic ear bars. Core temperature was monitored using a rectal thermistor and maintained at 37°C with a homeothermic blanket (Harvard Apparatus, Kent, UK). Aseptic surgical techniques were employed throughout.

#### Lumbar spinal exposure

The spinous process of T13 was identified by tracing the lowest rib to the vertebrae. Through a midline incision, the paraspinous muscles were detached from T12–L2 and a laminectomy was performed using fine rongeurs (Fine Science Tools, Heidelberg, Germany) to allow access to the spinal cord at L4–5. The spinous process of L3 was fixed in a spinal clamp (Narishige, Tokyo, Japan) to minimize movement during injections.

#### Cervical spinal exposure

The head of the rat was angled down (incisor bar at −20 mm) and through a midline incision the paraspinous muscles were parted to define the prominent T2 spinous process that was clamped and axially distracted. This allowed a laminectomy to be performed to expose the dorsal aspects of C6–C7 segments of the spinal cord.

#### Adenoviral vector injection

**Figure 1:**
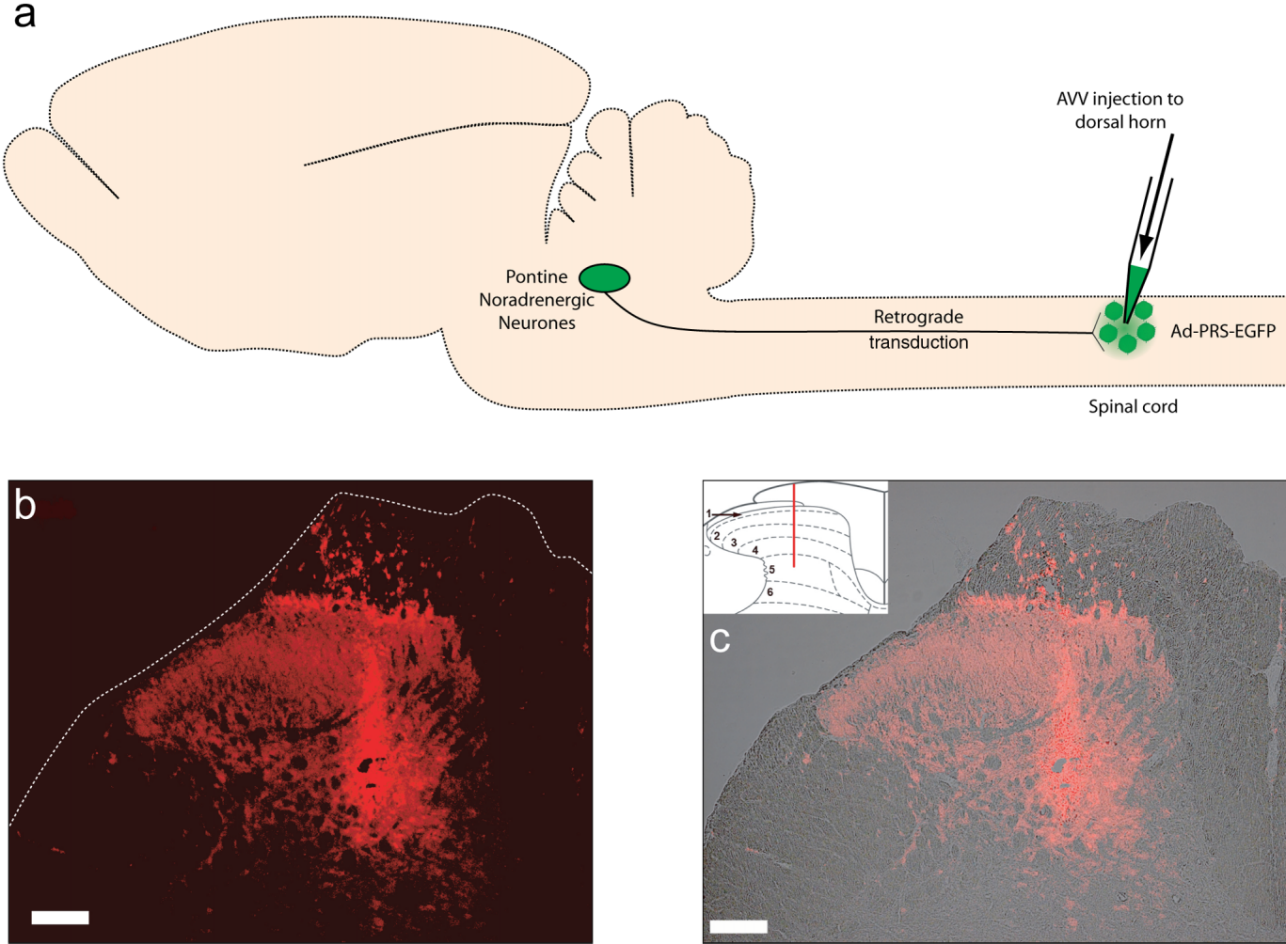
Retrograde targeting of brainstem noradrenergic neurons using spinal injection of AVV. **a**: Schematic of experimental approach. AVV-PRS-EGFP is injected into the dorsal horn of the lumbar spinal cord. The AVV is taken up by axon terminals of the pontospinal noradrenergic neurons and retrogradely transported. EGFP is expressed under the control of the catecholaminergic selective promoter PRS to enable visualization of the neuronal anatomy. **b**: The location and depth of the injection site is indicated by hexon protein-immunoreactivity (Cy3 fluorescence) in the spinal cord (1 day after unilateral AVV-injection). **c**: Overlaid brightfield and fluorescence images shows the spread of the AAV to be limited to the dorsal horn. Inset shows Rexed's laminae, red line indicates the injection trajectory. Scale bar = 100 μm.

Injections of AVV were made using a microcapillary pipette (calibrated in 1 μL intervals; Sigma, St. Louis, MO), with a tip diameter of around 20 μm. A 25 μL syringe (Hamilton, Bonaduz, Switzerland) was connected to the pipette with tubing primed with mineral oil. Using a syringe driver (Genie; Kent Scientific, Litchfield, CT) the pipette was backfilled with AVV. The pipette was directed into the dorsal horn using a micromanipulator (Narishige) to coordinates 400 μm lateral to the midline, 500 μm deep to dorsal surface. The successful delivery of AVV was visually confirmed on each occasion by observing the movement of the oil-vector medium interface in the calibrated capillary. Unless otherwise stated, two pairs of bilateral injections (each 500 μm apart in the rostrocaudal axis) of AVV (500 nL/injection over 2 minutes) were made into a single spinal segment (Fig. 1).

#### Recovery

The paraspinous muscles were sutured and the skin was closed with clips. Animals were given 1 mL sterile saline (i.p.) for fluid replacement, buprenorphine (2 μg / 100 g s.c., Temgesic, Schering-Plough, Hertfordshire, UK) for pain relief and atipamezole (an alpha2-adrenoceptor antagonist, 0.1 mg / 100 g i.p., Antisedan, Pfizer) to reverse the medetomidine anesthesia. Animals were monitored in a recovery room for the first 24 hours following surgery.

#### Tissue fixation

Rats were sacrificed (at 7 days, unless otherwise stated) with an overdose of pentobarbital (20 mg / 100 g i.p., Euthatal, Merial Animal Health, Essex, UK). After an intracardiac dose of heparin (100 IU) the animal was perfused transcardially with 0.9% NaCl (1 mL/g) followed by 4% formaldehyde (Sigma) in 0.1 M phosphate buffer (PB, pH 7.4, 1 mL/g). After perfusion the L4 and L5 dorsal root ganglia were identified, marked, and their roots used as a reference for segmental identification of the spinal cord. The brain and spinal cord were removed and postfixed for 2 hours before overnight cryoprotection in 30% sucrose in 0.1 M PB. To distinguish orientation the tissue was marked with a needle puncture site.

### Identification of spinal injection sites

The spinal cord from rats at 1–14 day post-AVV injection was cut in either coronal, longitudinal, or sagittal planes. The injection tracks were typically visible allowing confirmation of the location of the injection site within the target area of the dorsal horn. Immunocytochemistry for hexon protein, a component of the adenoviral coat, determined the extent of spread of the injected AVV. Examination of spinal cord tissue at the earliest timepoint (24 hours after the injection) indicated that the spread of AVV was confined to the ipsilateral dorsal horn (see Fig. 1). The expression of hexon viral protein was seen in a reduced distribution at day 3, and by day 7 only a “halo” region around the injection site (within 100 μm) was observed.

### Immunohistochemistry

Tissue sections were cut at 40-μm intervals using a freezing microtome and either serially mounted or left free-floating for fluorescence immunohistochemistry (IHC). Mounted sections were ringed with a DAKO pen (DAKO, Ely, UK) for on-slide incubations. Tissue sections were washed (×3) in 0.1 M phosphate-buffered 0.9% NaCl (pH 7.4, PBS) and permeabilized in 50% ethanol for 30 minutes before further washing. The tissue was incubated with primary antibodies (see Table 1) in PBS with 5% horse serum (HS), 0.3% Triton X-100, and 0.01% sodium azide for 24–72 hours at room temperature. Mounted sections were kept in a humidified chamber (RA Lamb, Eastbourne, UK) and free-floating sections were continuously agitated. After further washing, sections were incubated overnight with biotinylated secondary antibody (all Jackson ImmunoResearch, West Grove, PA; see Table 1) diluted 1:1,000 in PBS with 2% HS and 0.3% Triton. Sections were washed, incubated with Streptavidin Cy3 (1:1,000 in PBS, Sigma) for 4 hours before a final wash. Additionally some sections were incubated with DAPI (4′,6-diamidino-2-phenylindole dilactate, 1 μg/mL, Invitrogen, La Jolla, CA) for 15 minutes to delineate the nucleus. Controls were routinely run, by omitting primary antibodies, to ensure the specificity of immunostaining.

**Table 1:** Antibodies

Primaries				

Epitope	Dilution	Species	Source	Code/batch
Hexon	1:2,000	Goat	Biodesign International, Kennebunk, ME	B65101G/4A03106
DBH	1:10,000	Mouse	Chemicon, Temecula, CA	MAB308/24120144
c-fos	1:2,000	Rabbit	Santa Cruz Biotechnology, Santa Cruz, CA	sc-52/H0105
GFP	1:4,000	Rabbit	Invitrogen, Paisley, UK	A11122/56884A
CTb	1:2,000	Goat	List Biological Labs, Campbell, CA	703-2A5

Secondaries (raised in donkey)				

Anti-	Tag	Dilution	Source	Code

Mouse	AMCA	1:100	Jackson, West Grove, PA	715-156-150
Rabbit	Biotinylated	1:500-1,000	Jackson	711-066-152
Mouse	Biotinylated	1:1,000	Jackson	715-066-150
Goat	Biotinylated	1:1,000	Jackson	705-066-147
Goat	Alexa 594	1:200	Invitrogen	A-11058

For double labeling experiments to identify c-fos and DBH-immunoreactive (-ir) neurons, sections were incubated with anti-DBH (1:5,000) and anti-c-fos (1:2,000) in PBS with 5% HS, 0.3% Triton, and 0.01% sodium azide for 48 hours. Sections were washed before overnight incubation with AMCA-conjugated antimouse (1:100) and biotinylated antirabbit (1:500) secondaries in PBS with 2% HS and 0.3% Triton. After further washes, the tissue was incubated with Streptavidin Cy3 (1:500 in PBS) for 4 hours.

The mouse monoclonal anti-DBH antibody (Chemicon, Temecula, CA; MAB308) was raised against purified bovine DBH. The distribution of labeled neurons within the brainstem closely resembled that described for catecholaminergic neurons reported by previous investigators in the rat (Dahlstrom and Fuxe, 1964; Swanson, 1976; Grzanna and Molliver, 1980) and specifically in studies using this particular antibody (Rinaman, 2001; Espana and Berridge, 2006). In the absence of a commercial source of purified bovine DBH, antibody specificity was confirmed using a previously described preabsorption protocol (Dvoryanchikov et al., 2007). In brief, 1:10,000 dilutions of the anti-DBH antibody were preincubated in wells at room temperature either with or without a 40-μm section of fixed rat adrenal medulla. The aliquots were then aspirated and used in parallel according to the previously described IHC protocol against serial sections of pontine tissue containing the LC. The fluorescence labeling of LC neurons was abolished by the preincubation of antibody with the adrenal medulla.

The anti-c-fos polyclonal rabbit IgG (Santa Cruz Biologicals, Santa Cruz, CA; sc-52) was raised against a N-terminus peptide from human c-fos (amino acids 3–16, sc-52P). This antibody recognized a 62-kDa protein, inducible by phorbol ester application, on Western blot corresponding to the expected molecular weight of c-fos (manufacturer's technical datasheet). The immunoreactivity for c-fos induced in response to formalin testing was eliminated in both the spinal cord and the locus coeruleus by overnight preabsorption of the anti-c-fos antiserum with 0.1 μg/mL of the peptide antigen sc-52P.

The anti-GFP rabbit polyclonal IgG (Invitrogen, A11122) was raised against GFP protein extracted from *Aequorea victoria* and affinity column purified; its specificity has been confirmed in rat neurons transfected with GFP-expressing vectors (Card et al., 2006). In our control experiments no labeling was seen in brain tissue from animals that had not been transfected with AVV-PRS-EGFP.

The antihexon goat polyclonal IgG (Biodesign International, B65101G) was raised against hexon protein from adenovirus type 2 and also recognizes hexon from adenovirus types 5 and 6. It identifies a protein of 105 kDa on Western blot corresponding to the expected molecular mass of hexon protein (manufacturer's technical datasheet). Specificity of this antibody for our adenoviral vector was tested by preincubation of antihexon antibody (1:1,000) with Ad-PRS-EGFP (4 × 10^9^ TU/mL) overnight. Subsequent IHC using control and preabsorbed antihexon antibody (1:2,000) on serial sections of spinal cord injection sites (24 hours after injection of Ad-PRS-EGFP) showed that preabsorption eliminated the fluorescence labeling surrounding the injection site.

### Data analysis and photomicrography

All sections were mounted and coverslipped with fluorescent mounting medium (DAKO). Tissue was examined and representative images obtained using a conventional fluorescence microscope (Zeiss Axioskop 2) or with a confocal microscope (Leica DMIRBE and TCSNT). Images were acquired and processed using the respective company software (to adjust contrast and brightness) and, if required, images were further processed using Adobe Photoshop CS2 (Adobe Systems, San Jose, CA) to annotate structures and add scale bars.

Retrogradely labeled pontospinal noradrenergic neurons were counted as being present within each section when the nucleus could be identified within the cell body and a primary dendrite was visible. Counts of DBH-positive cells were made from noncontiguous sections (1-in-4). Using DAPI staining (n = 2 rats) we obtained an accurate estimate for the size of the nucleus in the retrogradely labeled NA neurons (9.6 ± 0.6 μm, n = 32) and used this average dimension to Abercrombie (1946) correct all NA neuron cell counts.

Data reported as mean ± SEM. Statistical significance was assessed using unpaired *t*-test or one-way ANOVA with Bonferroni's post-hoc test (Prism4, GraphPad, San Diego, CA), differences were considered significant at *P* < 0.05.

### Experimental protocols

#### Targeting noradrenergic neurons projecting to the lumbar dorsal horn

To test whether the AVV could retrogradely target the pontospinal noradrenergic neurons we injected AVV-PRS-EGFP (3 × 10^10^ TU/mL) bilaterally into the lumbar (L4–5) dorsal horn of spinal cord (n = 14). The spinal cord, brainstem, and forebrain were examined for the presence of EGFP-positive neurons (the morphology of the cell somata was examined in detail in nine of these animals). DBH IHC was used to examine whether the EGFP expression was restricted to catecholaminergic neurons (n = 6). An indication of the relative contributions of each of the pontine NA nuclei to the pontospinal projection was obtained from counts of DBH-positive neurons in each region. These counts were determined directly for the A5 and A7 regions (n = 3 rats) and we used previously obtained values for the LC (Loughlin et al., 1986). This allowed the calculation of the proportion of neurons in each region that projected to the lumbar spinal cord.

We compared AVV-PRS-EGFP with the conventional retrograde tracers FluoroGold (FG, Fluorochrome, Denver, CO), cholera toxin b subunit (CTb, List Biological Laboratories, Campbell, CA), and red fluorescent latex microspheres (Retrobeads, Lumafluor, Naples, FL). For these experiments AVV (3 × 10^10^ TU/mL) was injected (at two sites bilaterally) into the L4/L5 spinal cord (n = 9 rats). To obtain an estimate of the size of the total population of NA neurons projecting to the lumbar spinal cord a pair of FG (5%, 50 nL each side, n = 2 rats) or CTb (1%, 500 nL, n = 4 rats) injections was made into the spinal dorsal horn, interleaved between the AVV injections. We also made use of fluorescent microspheres, which are known to have a more restricted distribution from the injections site to identify the NA neurons projecting specifically to the L4–5 dorsal horn (100 nL, at two sites bilaterally) interleaved with the AVV injections in the rostrocaudal axis (n = 3 rats). One further animal received co-injections (500 nL, two sites bilaterally) of a mixture of AAV-PRS-EGFP (3 × 10^10^ TU/mL) and red microspheres (1:2 dilution). For the CTb injections retrogradely labeled brainstem neurons were detected by IHC for CTb (1:2,000, 3 days at 4°C, goat anti-CTb, List Biological Laboratories) and revealed by incubation with fluorescent secondary antibody (antigoat Alexa 594, 1:200, overnight, Invitrogen). All brainstem tissue was processed for DBH IHC to determine the numbers of noradrenergic neurons labeled by FG/CTb/beads.

#### Factors influencing retrograde viral transduction

##### Effect of AVV titer

AVV-PRS-EGFP was diluted with PBS prior to spinal injection to obtain titers of 3, 30, 100 (n = 3 per group), 300 (n = 14), and 1000 × 10^8^ (n = 3) TU/mL.

##### Influence of time postinjection on retrograde labeling

AVV-PRS-EGFP (3 × 10^10^ TU/mL) was injected into L4/L5 of the dorsal horn. Rats (n = 31) were divided into five groups and culled at the following timepoints after injection: day 1 (24 hours, n = 3), day 2 (n = 3), day 5 (n = 3), day 7 (n = 14), and day 14 (n = 8).

##### AVV injection into lumbar cerebrospinal fluid

To test whether intraparenchymal delivery was required for retrograde labeling AVV-PRS-EGFP was injected intrathecally into the cerebrospinal fluid, over the lumbar dorsal horn (3 × 10^10^ TU/mL, 2 μL, n = 2).

#### Topographical organization of the pontospinal noradrenergic projection

Injections of AVV-PRS-EGFP or -mRFP were made into discrete locations within the spinal cord to examine the topography of the pontospinal NA projection.

##### Spinal segmental organization of the noradrenergic projection

One group of rats (n = 4) had a two-level laminectomy (L4–5 and C6–7) and received bilateral injections of AVV-PRS-EGFP or AVV-PRS-mRFP (1.2 × 10^10^ TU/mL) into L4/L5 and C6/C7 spinal segments (respectively). The control group (n = 3) had bilateral injections placed into the L4/5 spinal segments, of both AVV-PRS-EGFP and AVV-PRS-mRFP (both 1.2 × 10^10^ TU/mL). The injection sites for each AVV were interleaved within the same segment so that there was a rostrocaudal distance of 250 μm between successive injections sites (i.e., 500 μm between consecutive AVV-PRS-EGFP injection sites). We also examined the effect of bilateral co-injection of AVV-PRS-EGFP and AVV-PRS-mRFP (both 8 × 10^9^ TU/mL, mixed in the same pipette) to the L4/L5 dorsal horn (n = 3).

Animals were culled 14 days after AVV injection. Retrogradely labeled brainstem neurons were counted and the proportion of double-labeled cells was expressed as a percentage of the total number of mRFP-positive neurons.

##### Lateralization of pontospinal noradrenergic projection

Rats (n = 3) received four unilateral injections of AVV-PRS-EGFP (3 × 10^10^ TU/mL) into L4/L5 dorsal horn. The distribution of retrogradely labeled neurons (ipsi- vs. contralateral) was expressed as percentage of the total number of neurons in the A5, LC, and A7 groups.

#### Projections of pontospinal NA neurons to other brain regions

The extent of the axonal arbor of the pontospinal NA neurons that innervate dorsal horn of the L4/L5 spinal segments were revealed using anti-EGFP IHC. Rats (n = 5) had bilateral spinal injections of AVV-PRS-EGFP (3 × 10^10^ TU/mL) and were culled 14 days later. Brain tissue was cut in the coronal plane, while spinal cord tissue was cut in coronal, longitudinal, and sagittal planes. To contrast the pattern of distribution of noradrenergic fibers with the EGFP containing projections from pontospinal neurons, alternate sections were processed for anti-GFP and anti-DBH IHC (in two animals). The density of the EGFP containing fiber projections revealed by anti-GFP IHC was scored for each animal on a rating scale from 0 to 5, with 0 representing a total absence of fibers to 5 being densest (e.g., principal nucleus of the olive).

To test whether the pontospinal NA neurons had multiple projection targets a further two animals received both lumbar spinal injections of AVV-PRS-EGFP and injections of red fluorescent beads (four unilateral injections of 100 nL) into sites within the ventral posterolateral thalamic nucleus (micropipette lowered vertically through a craniotomy burr hole to injection sites located between bregma −2.3 to −3.5 mm, ML 3–3.6 mm, and depth 6–6.4 mm; coordinates after Paxinos and Watson, 2005). Animals were culled 14 days after AVV and bead injections. The bead injection sites were confirmed as being in the thalamic VPL and the numbers of retrogradely labeled neurons were counted in A5, LC, and A7 regions. The proportion of bead and EGFP double-labeled cells was expressed as percentage of the total number of EGFP-positive neurons.

#### Activation of pontospinal NA neurons during the formalin test

All animals were handled daily and habituated to the testing room, experimental equipment, and handlers. Rats (n = 10) received bilateral injections of AVV-PRS-EGFP (3 × 10^10^ TU/mL) into the L4/L5 dorsal horn. One week later the animals were divided into two groups for the formalin test, a test group (n = 8) and a control group (n = 2). Rats were acclimatized for 30 minutes to a clear Plexiglas testing chamber before either formalin (5% neutral buffered) or NaCl (0.9%) was injected subcutaneously (50 μL, 30G needle) to raise a bleb on the dorsal surface of the right hindpaw.

Rats were replaced in the testing chamber and the numbers of flinches and foot lifts were tallied over 1-minute periods, initially every 2 minutes for the first 10 minutes and then every 5 minutes for the remainder of the 60 minutes. Animals were culled 2 hours after the end of the observation period to allow optimal c-fos expression. Brainstem tissue was processed for c-fos and DBH IHC in all rats and counts were made of retrogradely labeled pontospinal neurons showing c-fos expression across each cell group. In addition, spinal c-fos expression was quantified (control [n = 2] and formalin test rats [n = 3]) by tallying the positive neurons from 10 coronal, nonsequential, spinal cord sections (40 μm) from L4–5 with the greatest numbers of c-fos positive nuclei. Comparisons were made between c-fos counts in control and test group animals and between ipsi- and contralateral dorsal horns.

## RESULTS

### Targeting noradrenergic neurons projecting to the lumbar dorsal horn

#### Distribution of cell somata

**Figure 2:**
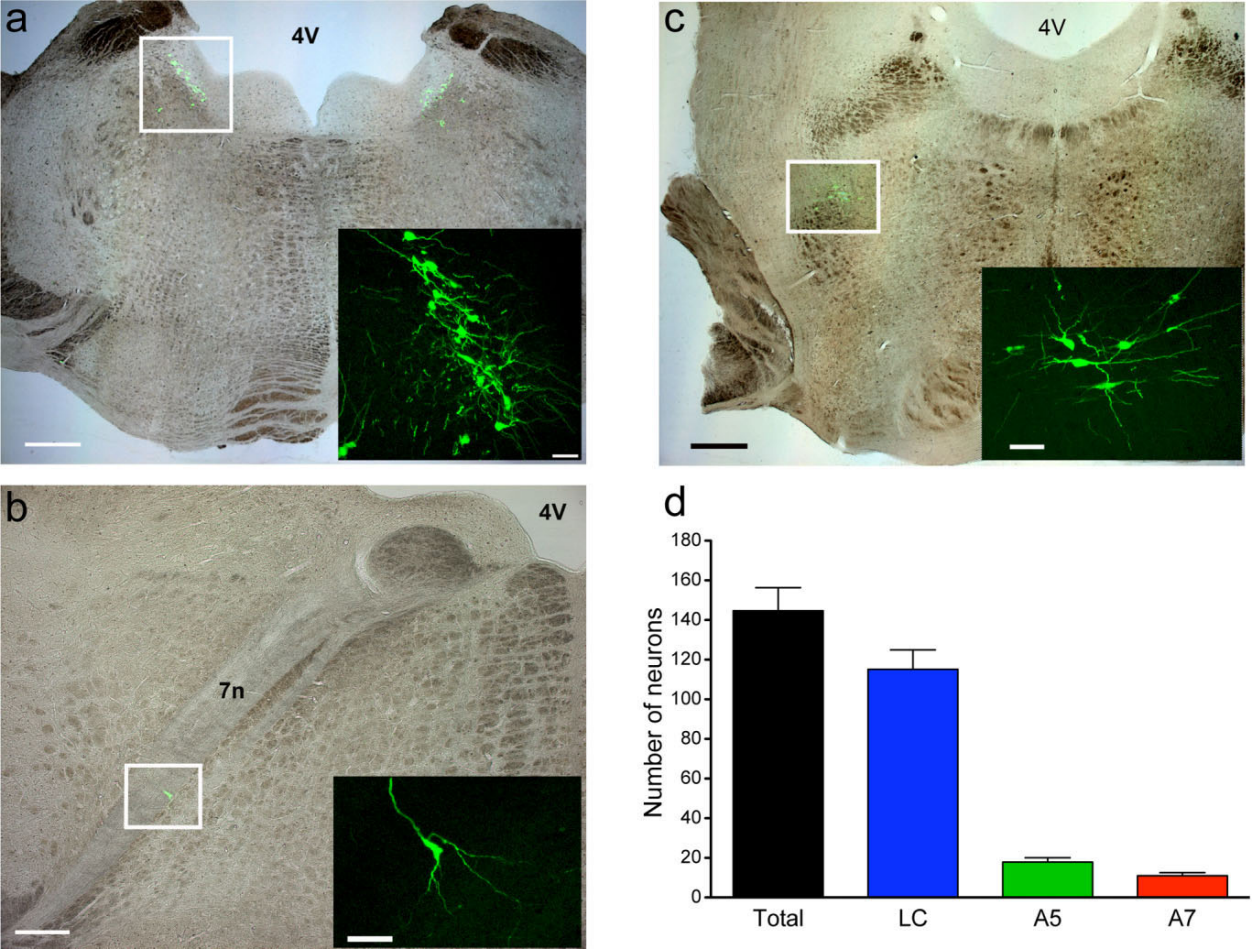
Retrograde labeling of pontospinal neurons in A5, LC, and A7. Retrogradely labeled pontine neurons following transduction of the L4-5 dorsal horn with AVV-PRS-EGFP. Fluorescent neurons are seen in locus coeruleus (**a**), A5 (**b**), and A7 (**c**) regions. Overlaid brightfield and fluorescence images (a–c) with inset overlaid fluorescence confocal stacks. **d**: Distribution of pontospinal neurons projecting to L4–5 dorsal horn. Following administration of AVV-PRS-EGFP to the lumbar dorsal horn an average of 145 ± 12 (n = 14) retrogradely labeled neurons were found in the pons. The majority of the cell somata were found in the locus coeruleus with the remainder in A5 and A7 clusters. (Data shown mean ± SEM). 4V, 4th ventricle, 7n, facial tract. Scale bars = 1,000 μm in a,c; 500 μm in b; 80 μm in all insets.

Following spinal injection of AVV-PRS-EGFP (3 × 10^10^ TU/mL) into L4–L5 dorsal horn, retrogradely labeled fluorescent (EGFP-expressing) neurons were only seen in the pontine A5, LC, and A7 areas (Fig. 2). The total number of labeled pontine neurons at 7 days postinjection was 145 ± 12 (n = 14) with a majority in LC (115 ± 10; 80%) and the remainder in A5 (18 ± 2; 12%) and A7 (11 ± 2; 8%, Fig. 2d). Labeled somata were scattered throughout the LC, but with a higher density in the ventral pole of the nucleus. The strength of EGFP expression in the pontospinal neurons allowed clear visualization of the soma and proximal dendritic arbor. Examination of LC cell morphology (from nine rats) showed the majority of retrogradely labeled somata were fusiform (70 ± 3%), with the remainder being multipolar (24 ± 3%) or round (6 ± 1%).

**Figure 3:**
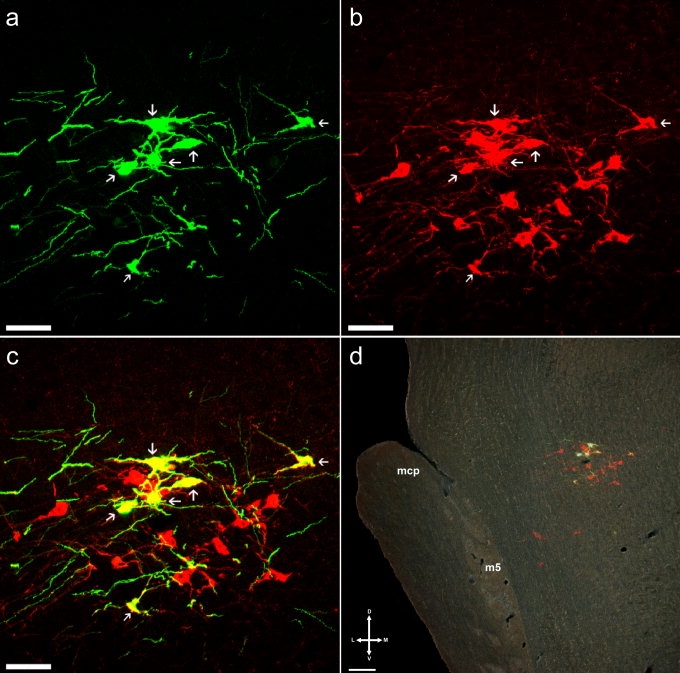
Retrogradely labeled neurons are catecholaminergic. Confocal stack images showing (**a**) EGFP-positive retrogradely labeled neurons in rostral A7 region, (**b**) DBH-ir showing the cluster of A7 neurons (Cy3 red fluorescence), and (**c**) overlay of confocal stack images confirming all EGFP-positive neurons are DBH-positive (**d**) darkfield image with overlaid fluorescence showing relative position of A7 neurons (bregma −8.4 mm). m5, motor root of the trigeminal nerve, mcp, middle cerebellar peduncle. Magenta-green copy available as Supplementary Figure 1. Scale bars = 80 μm in a–c; 100 μm in d.

The retrogradely labeled neurons showed DBH-ir (A5, 97 ± 3%; LC, 97 ± 4%; and A7, 100 ± 0%, n = 6, see Fig. 3), indicating that the PRS promoter restricted the expression of EGFP to catecholaminergic neurons. The retrogradely labeled LC neurons that we were unable to unequivocally determine as being DBH-ir (1.6%, 10/622, n = 6 rats) were found in the core of the nucleus and had morphologies consistent with being noradrenergic. However, because of the density of DBH staining seen in the closely packed cells in the core of the LC it was occasionally difficult to distinguish unequivocally between low fluorescence signal in the labeled cell and that from adjacent somata. Given the location of these cells within the core of the LC, it would seem likely that they represent NA neurons with faint DBH-ir.

The A5 and A7 regions were identified using the Paxinos and Watson atlas (2005) and the total numbers of DBH-ir neurons were counted in A5 (1553 ± 337, n = 3) and A7 (346 ± 43, n = 3). The number of DBH-positive neurons in the LC (3268 ± 114) was taken from previous work (Loughlin et al., 1986). Hence, it was possible to determine the proportionate distribution of somata retrogradely labeled from L4–5 in each of these cell groups expressed as a percentage of the total number of NA neurons in each area (A5, 1.4 ± 0.2%; LC, 4.4 ± 0.4%; and A7, 4.0 ± 0.6%, n = 14 rats).

Spinal co-injections of AVV-PRS-EGFP and FluoroGold (FG) or CTb were used to compare the viral vector with conventional retrograde tracers. Both FG and CTb demonstrated numerous retrogradely labeled neurons throughout the brainstem, including DBH-positive neurons in the A5, LC (predominately ventral) and A7 regions (FG: A5, 184 ± 37, LC, 244 ± 8, A7, 61 ± 10, n = 2 rats; CTb: A5, 68 ± 18, LC, 161 ± 51, A7, 47 ± 21, n = 4 rats) and also in the rostral ventrolateral medulla. Examination of the spinal cord after injection revealed an extensive spread of both FG and CTb from the site of injection with dispersion over many millimeters in the rostrocaudal axis and extension through the full thickness of the cord to the ventral horn. This extensive spread of tracer would be expected to have labeled neurons projecting to most of the lumbar spinal enlargement as compared to the AVV, which had more spread within restricted to the dorsal horn of two segments (to a 300 μm radius, Fig. 1). It should also be noted that there was no pontine EGFP expression seen in the FG/CTb experiments, indicating an apparent incompatibility in the use of these conventional tracers with our adenoviral vector (as suggested previously for FG: Schramm, 2006).

The spinally injected red latex microspheres showed a similar restricted distribution in the cord to the AVV-PRS-EGFP (n = 3). Within the pontine NA nuclei the beads and AVV-PRS-EGFP labeled similar numbers of neurons (78 ± 25 vs. 110 ± 49 respectively, n = 3, NS). Most of the bead-positive neurons in the A5, LC, and A7 areas were DBH-positive (82%). The distribution of bead-positive neurons across the NA cell groups was essentially identical to that seen with the viral vector with most located in the LC (79%) and the remainder in A5 (15%) and A7 (5%). The interleaved injections (n = 3 rats) showed 18 ± 4% double labeling of EGFP neurons with beads. By contrast the co-injection of AVV with beads showed 60% colocalization (n = 1 rat, note there was an apparent loss of efficacy of AVV labeling with this co-injection approach with only 10 neurons being EGFP-positive).

### Factors influencing retrograde AVV transduction

#### Effect of AVV titer

**Figure 4:**
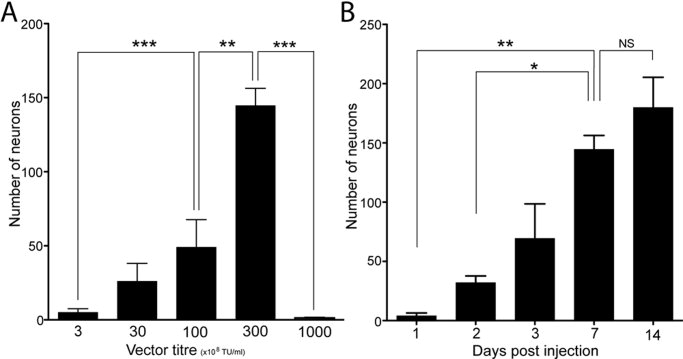
Factors influencing efficiency of retrograde labeling. **a**: The titer of AVV-PRS-EGFP injected to the spinal cord was varied from 3–1,000 × 10^8^ TU/mL (n = 3 in each group, except 300 where n = 14). Efficient retrograde labeling required higher viral titers (>100 × 10^8^ TU/mL). However, at the highest AVV titer (1,000 × 10^8^ TU/mL) the numbers of EGFP-positive neurons fell sharply. **b**: Time-course of retrograde transduction by AVV-PRS-EGFP was examined by varying the duration of survival following spinal AVV injection. Significant numbers of fluorescent retrogradely labeled pontine neurons were first seen at day 2 after AVV injection and there was a steady increase in the numbers until day 7, when a plateau was reached (n = 3 for each group except day 7 [n = 14] and day 14 [n = 8]). (Means ± SEM, one-way ANOVA with Bonferroni's multiple comparison test, *P < 0.05, **P < 0.01, ***P < 0.0001.)

The efficiency of retrograde labeling was dependent on the titer of AVV injected into the spinal cord. Serial dilution of the AVV injectate produced reductions in the numbers of retrogradely labeled neurons (see Fig. 4a) from 145 ± 12 (300 × 10^8^ TU/mL, control) to 49 ± 19 (at 100 × 10^8^, *P* < 0.0001) and down to as few as 5 ± 3 (at 3 × 10^8^ TU/mL). Interestingly, at the highest AVV titers (1,000 × 10^8^ TU/mL) there was a dramatic fall in the numbers of retrogradely labeled neurons.

#### Influence of time postinjection

The first pontine retrograde labeling could be seen 24–48 hours after spinal injection of AVV-PRS-EGFP (Fig. 4b). The numbers of labeled pontine neurons increased with longer expression periods up to day 7, when it peaked and no further increase in the numbers of EGFP-positive neurons was seen at day 14. The visible extent of the dendritic tree of labeled neurons appeared greater at day 14 compared to that seen at earlier timepoints, suggesting the presence of higher concentrations of EGFP in the processes.

#### AVV injection into lumbar cerebrospinal fluid

One week after intrathecal injection of AVV-PRS-EGFP (2 μL, 3 × 10^10^ TU/mL, n = 2 rats) no EGFP-positive neurons were seen in the pons or elsewhere in the brain or spinal cord, indicating that intraparenchymal spinal injection of this AVV is needed for retrograde labeling and importantly that the pontine labeling is not a consequence of rostral spread of vector carried in the CSF.

### Topographical organization of the pontospinal noradrenergic projection

**Figure 5:**
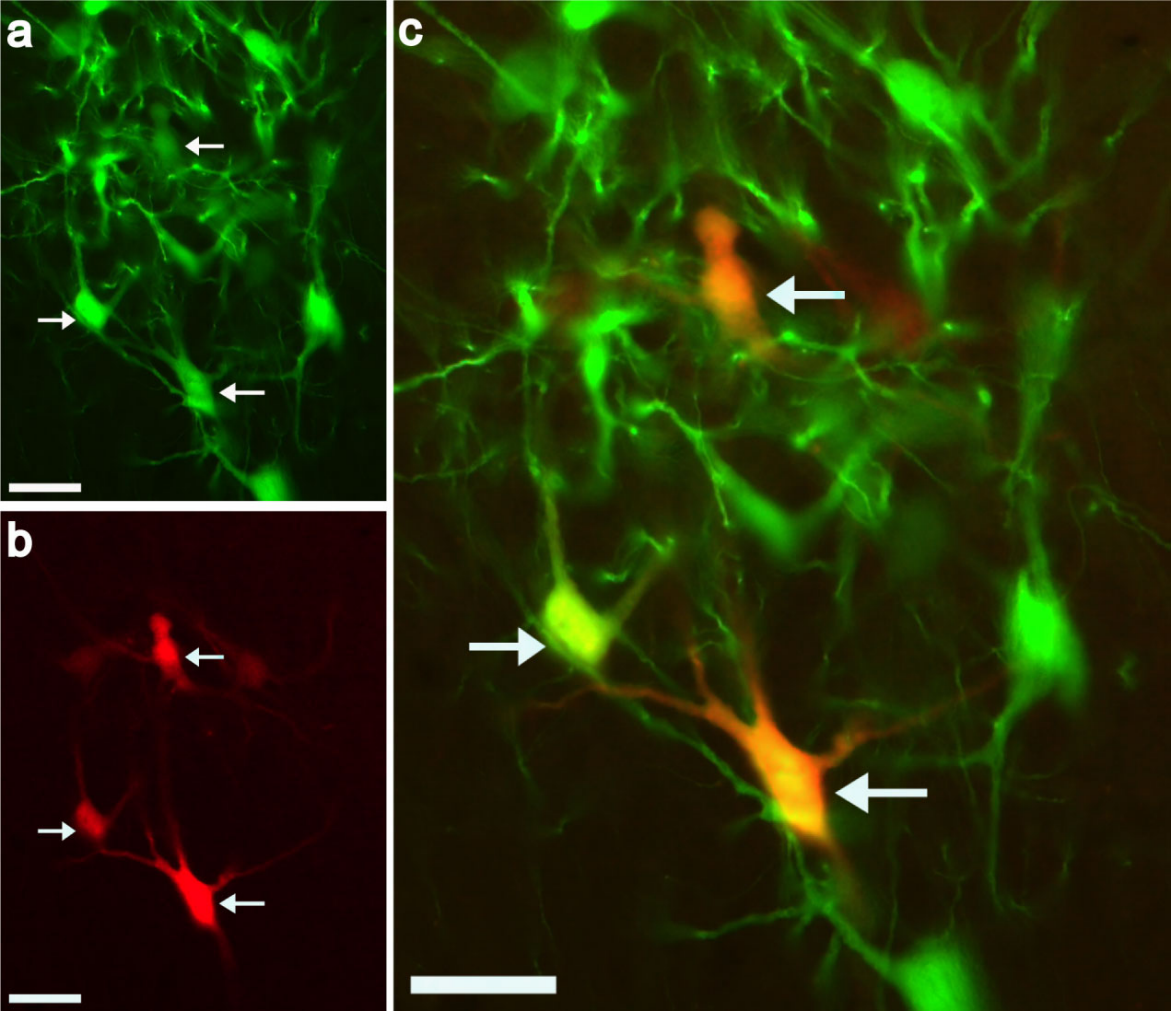
Locus coeruleus neurons with bilateral projections to the spinal cord. Ipsi- (AVV-PRS-EGFP) and contra- (AVV-PRS-mRFP) lateral injections of AVV and were made into the dorsal horn of L4/L5 to examine the topography of the projection of the pontospinal NA neurons. The colocalization of fluorophores (**a**) EGFP and (**b**) mRFP indicated neurons which projected bilaterally (white arrows, 4% of total) from the locus coeruleus to the spinal cord ((**c**), overlay). Magenta-green copy available as Supplementary Figure 2. Scale bars = 50 μm.

Unilateral spinal injection of AVV demonstrated a bilateral projection from the LC with an ipsilateral predominance (62 ± 4% of the labeled pontospinal NA neurons being ipsilateral, n = 3 rats; *P* < 0.05, unpaired *t*-test). Some of the pontospinal neurons (4%) were seen to project bilaterally to the lumbar dorsal horn using ipsi- and contralateral injections of AVV expressing EGFP or mRFP (see Fig. 5).

#### Spinal segmental organization of noradrenergic projection

The spinal segmental organization of the NA projection was examined using injections of AVV-PRS-EGFP and AVV-PRS-mRFP into the dorsal horn at different spinal levels (C6–7 and L4–5). This showed sparse double labeling of neurons, with only 1.3 ± 1.0% of mRFP-positive LC neurons also expressing EGFP (across all the pontospinal NA neurons only 1.0 ± 0.8% showed colocalization, n = 4 rats). This contrasted with the 18.6 ± 1.6% colocalization seen (n = 3; *P* < 0.0001) in the comparison group that received injections of each AVV placed into adjacent sites (placed 250 μm apart) within the L4/L5 segment. Also, lumbar spinal co-injection from a single pipette of AVV-PRS-EGFP and AVV-PRS-mRFP, mixed in equal titers, showed that 62 ± 3% (n = 3) of all mRFP-positive LC neurons expressed EGFP.

### Axonal projections of pontospinal NA neurons

**Figure 6:**
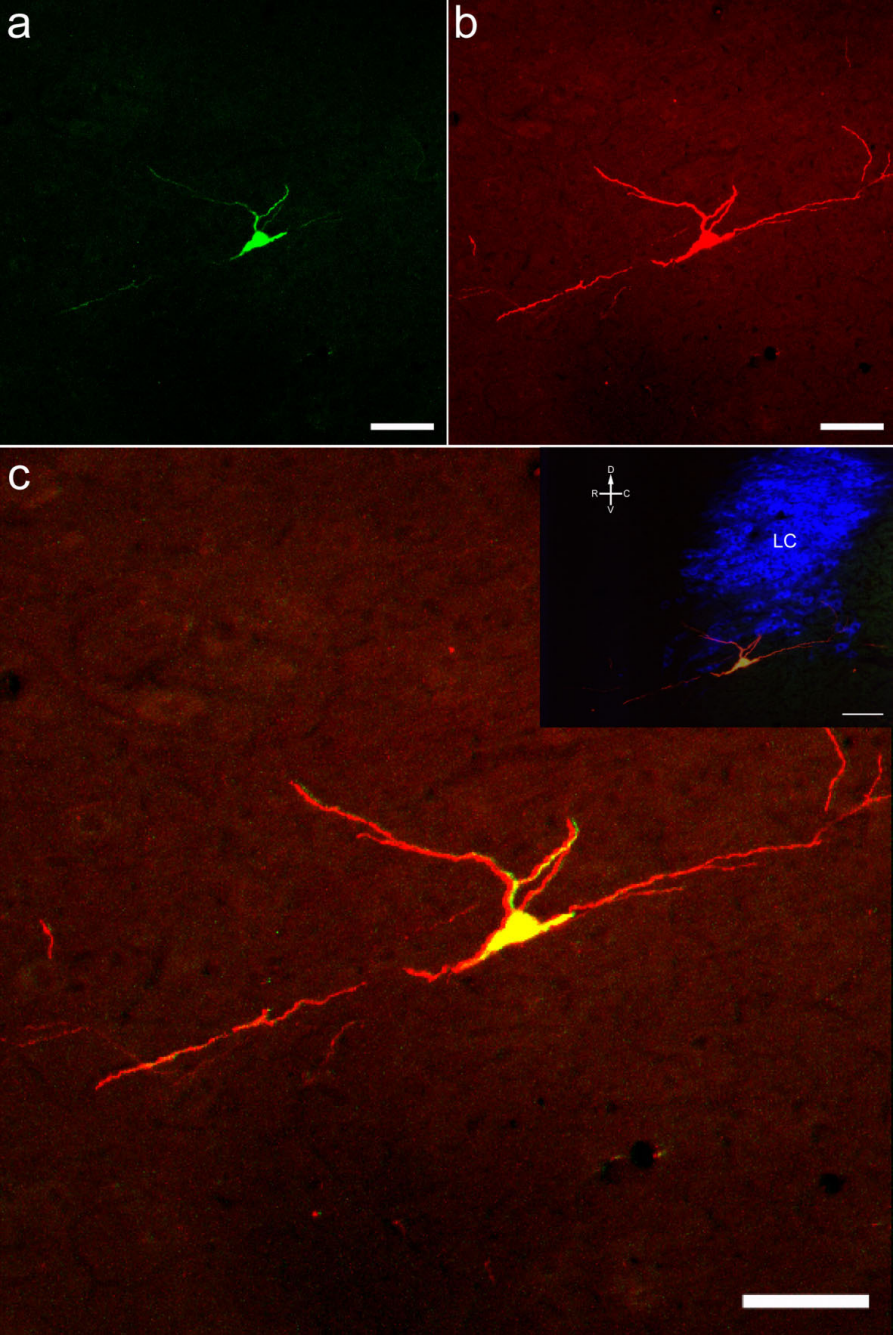
Demonstration of fine processes. Confocal stack images of a sagittal section showing an EGFP-positive neuron in the ventral pole of the LC (**a**) native EGFP fluorescence (**b**) GFP immunoreactivity (Cy3 labeling) produced an enhanced visualization of the fine dendritic and axonal processes of the neuron (**c**) overlay image; inset shows position of neuron within LC (DBH-ir AMCA, blue). Magenta-green copy available as Supplementary Figure 3. Scale bars = 80 μm, inset 100 μm.

**Figure 7:**
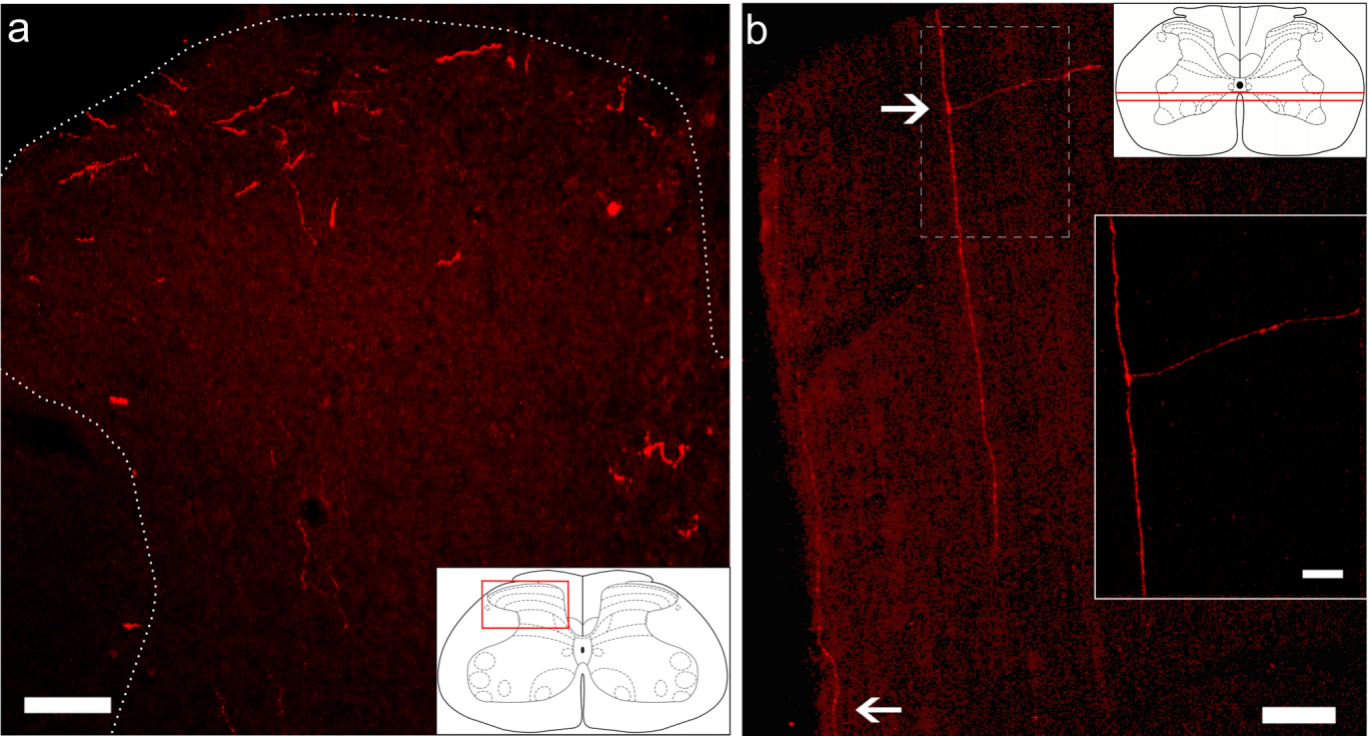
Axonal labeling in spinal cord. In the spinal cord, terminal labeling was revealed using anti-GFP IHC (Cy3 fluorophore, red). **a**: Lumbar dorsal horn showing a predominance in the superficial dorsal horn. **b**: Longitudinal sections (plane indicated by lines on inset) revealed axons projecting in the lateral and ventral funiculi (white arrows). Bifurcations were occasionally observed projecting into the gray matter of the spinal cord. Scale bars = 80 μm, inset 20 μm.

Using GFP IHC it was possible to amplify the EGFP fluorescence signal and reveal fine, distal processes (including axon terminal fields) of the retrogradely labeled pontospinal NA neurons throughout the neuroaxis (Figs. 6, 7; Table 2 for more details). DBH IHC allowed comparisons to be made with the overall distribution of NA projections in the brain and spinal cord and confirmed the GFP containing fibers to be DBH-positive.

**Table 2:** Axonal Projection Targets of Pontospinal Noradrenergic Neurons

Brain region	Area	Density
Neocortex	Cingulate	3
	Frontal	3
	Insular	3
	Piriform	3
	Entorhinal	1
Striatum	Globus pallidus	3
	Caudate putamen	1
	Nucleus accumbens	1
Hippocampus	CA1-CA3	0
	Dentate gyrus	0
Basal	Ventral pallidum	1
Forebrain		
Thalamus	Anteroventral nucleus	5
	Anterodorsal nucleus	5
	Mediodorsal nucleus	3
	Reticular nucleus	5
	Ventrolateral nucleus	5
	Ventroposterior nucleus (medial and lateral)	5
	Paratenial nucleus	2
	Habenular nuclei	3
Hypothalamus	Medial wall of paraventricular nucleus	3
	Lateral hypothalamus	3
Cerebellum	Molecular layer	3
	Granular layer	3
Midbrain	Periaqueductal gray	4
Hindbrain	Pontine Nuclei	5
	Parabrachial nucleus	3
	Inferior olive	
	-principal nucleus	5
	-medial nucleus	2
	-dorsal nucleus	2
Spinal Cord	Lumbar (L4-5)	
	-SDH (laminae 1-2)	5
	-DDH (laminae 3-6)	3
	-VH (laminae 7-9)	3
	-Central canal (Area X)	4
	-Ventral funiculus	4
	-Lateral funiculus	2
	-Gracile funiculus	2

Distribution of projections from pontospinal noradrenergic neurons demonstrated with GFP-ir. Density of GFP-ir fibres in each region on scale 0-5 (where 0=absent, 1=sparse and 5=dense).

#### Spinal cord

In the spinal cord, axonal projections, fine axonal branches, and terminal fields (Fig. 7a; Table 2) could be observed. In transverse sections of L4/L5, there was dense GFP-ir in the superficial dorsal horn (SDH, laminae I and II), moderate density of axonal projections in the deep dorsal horn (DDH, laminae III–V) and the ventral horn (laminae VII–IX). Moderate to dense labeling was observed around the central canal (area X). The densest GFP-ir labeling in the white matter of the spinal cord was found in the ventral funiculus, with light to moderate labeling in the lateral funiculus and gracile fasciculus. This pattern was confirmed in the longitudinal and sagittal sections, where long segments of GFP-ir axons were observed running close to ventral, lateral, and dorsal surfaces, with the majority observed in the ventral funiculus. Occasionally, these axons could be seen to send collaterals at right angles to project into the gray matter (see Fig. 7b).

#### Brain

In the telencephalon, light to moderate GFP-ir was observed in the neocortex (particularly in the cingulate, frontal, insula, and piriform areas) and globus pallidus. By contrast, relatively few axonal projections were observed in the hippocampus, caudate putamen, nucleus accumbens, and entorhinal cortex despite strong DBH-ir labeling. In the diencephalon the highest density of GFP-ir was observed in thalamus. However, in the hypothalamus only light to moderate GFP-ir was observed, in contrast with the strong DBH-ir.

In the cerebellum there was a moderate density of GFP-ir labeling compared to the denser DBH labeling. The brainstem showed moderate to dense GFP-ir staining, especially in the inferior olive, pontine nuclei, periaqueductal gray, paramedian raphe, ventral tegmental area, deep mesencephalic, and anterior pretectal nucleus. The GFP-ir in the inferior olive was strongest in the principal nucleus.

**Figure 8:**
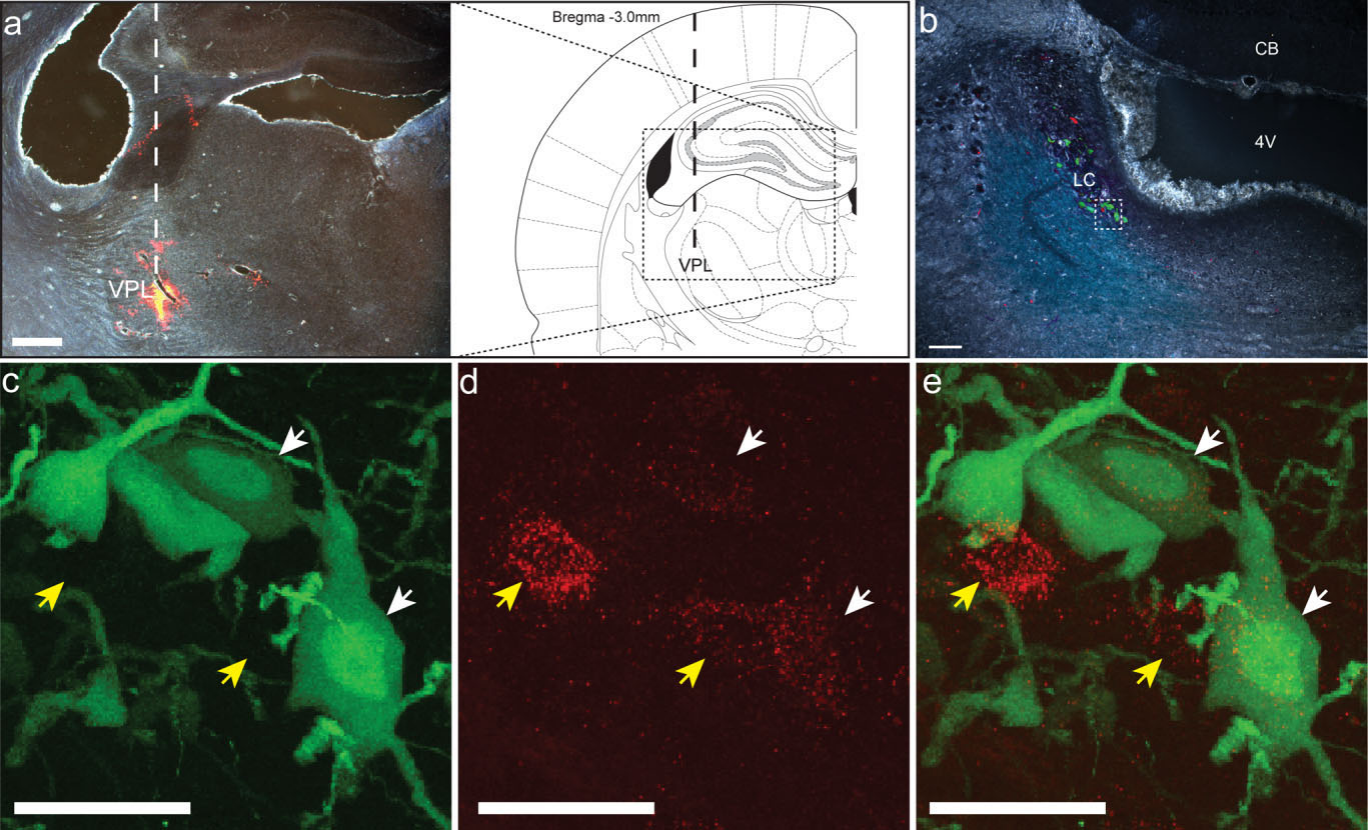
Pontospinal NA neurons can be double-labeled from the ventral posterolateral thalamic nucleus. Animals received microinjections of AVV-EGFP to the dorsal horn at L4/L5 and red fluorescent beads (4 × 100 nL) targeted to the thalamic VPL. **a**: Representative VPL injection site (overlaid fluorescence and darkfield image) and schematic (adapted from Paxinos and Watson, 2005) showing injection track (dashed line). **b**: Within the LC (shown overlaid on darkfield image) there were numerous labeled neurons identified retrogradely from spinal cord (EGFP) and/or thalamus (red beads). **c**–**e**: Confocal stack images of the LC (from boxed area in b) showing examples of retrogradely labeled EGFP-positive and red beaded neurons (arrows), with two neurons showing colocalization (e, white arrows). Magenta-green copy available as Supplementary Figure 4. VPL, ventral posterolateral nucleus; LC, locus coeruleus; CB, cerebellum; 4V, 4th ventricle. Scale bars = 500 μm in a,b; 25 μm in c–e.

To test whether these divergent projections originated from the pontospinal NA neurons we combined spinal AVV-PRS-EGFP with injections of red fluorescent beads into the VPL nucleus of the thalamus, a territory that we had noted to have a dense projection of EGFP-containing fibers. These thalamic injections retrogradely labeled neurons in the core of the LC and double labeling with beads was seen in 26 ± 3% of the EGFP-positive pontospinal NA neurons (n = 2, Fig. 8). No retrograde bead labeling at all was seen in the A7 region and few neurons were seen in the A5 region with no double labeling of pontospinal neurons.

### Activation of pontine-lumbospinal NA neurons during noxious stimuli/formalin test

**Figure 9:**
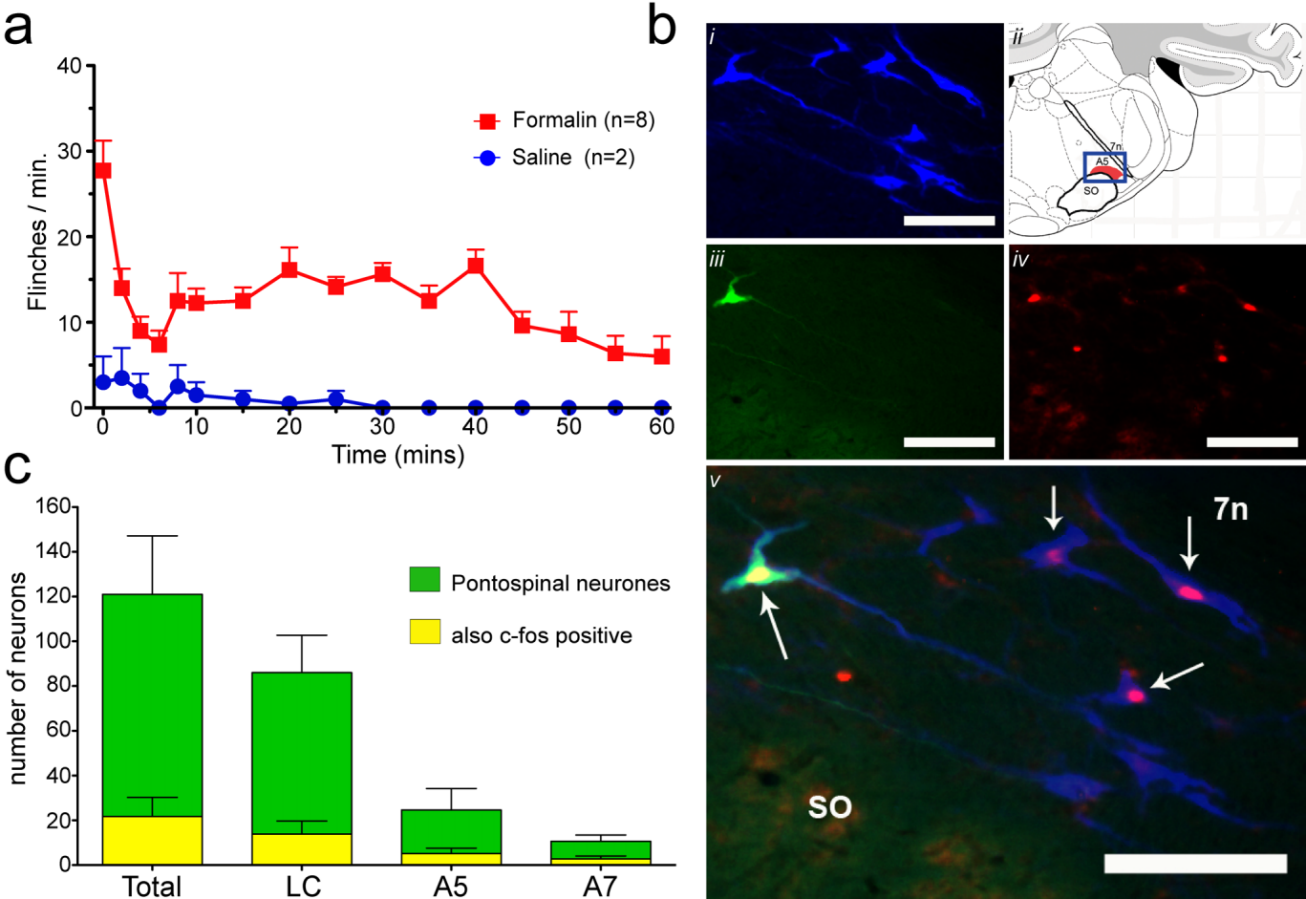
Activation of pontine lumbospinal NA neurons during the formalin test. **a**: Assessment of the nociceptive behavioral response to formalin (red squares) or control saline (blue circles) injected to the hindpaw in rats transfected with spinal AVV-PRS-EGFP. The response was quantified by counting the number of flinches and foot lifts each minute (every 2 minutes for the first 10 minutes, then every 5 minutes for the remainder of the test). Transfected animals showed typical biphasic behavioral response to the formalin injection. **b**: Pontine-lumbospinal NA neurons express c-fos following the formalin test. After the completion of the formalin test pontine tissue was processed to reveal c-fos-ir and DBH-ir. (i) A5 neurons revealed by the DBH stain (AMCA, blue). (ii) Annotated figure indicating location of cells (blue box, bregma −10.32 mm; Paxinos and Watson, 2005). (iii) One of the A5 neurons is retrogradely labeled (EGFP). (iv) Several neurons show nuclear c-fos-ir (Cy3, red) and from the overlay. (v) It is seen that the pontospinal neuron and several other DBH-positive neurons (white arrows), are c-fos-positive. 7n, facial nerve tract, SO, superior olive. **c**: Distribution of pontine-lumbospinal NA neurons expressing c-fos following the formalin test. Although the majority of c-fos-positive neurons were located in LC the proportional distribution was relatively even across the cell groups (A5, 21%, LC, 16%, and A7, 26%, n = 8). Scale bars = 100 μm.

Rats transfected with AVV-PRS-EGFP showed typical biphasic nociceptive behavioral responses (flinching, paw lifting, and licking) to formalin testing (n = 8 rats, see Fig. 9a). Injection of formalin compared to saline produced an ipsilateral increase in the expression of nuclear c-fos in the superficial (140 ± 55 [n = 3] vs. 1 ± 1 [n = 2] positive nuclei per 10 sections, respectively) and deep dorsal horn (135 ± 66 [n = 3] vs. 1 ± 1 [n = 2] positive nuclei per 10 sections, respectively). Formalin increased the brainstem expression of c-fos-ir in the periaqueductal gray, the parabrachial nucleus, as well as in the pontine noradrenergic cell groups (n = 8). Significantly more of the retrogradely labeled pontospinal NA neurons showed c-fos-ir expression after formalin compared to saline control (18% vs. <1% of neurons, n = 8 and 2 rats, respectively, *P* < 0.001, see Fig. 9b). The proportions of c-fos-positive pontospinal NA neurons were relatively similar across all of the pontine cell groups (A5, 21%, LC, 16%, and A7, 26%, n = 8, see Fig. 9c).

## DISCUSSION

In this study we demonstrated the ability of an AVV containing a catecholaminergic cell-specific promoter to selectively retrogradely target pontospinal noradrenergic neurons projecting to the lumbar dorsal horn. This retrograde targeting from the lumbar L4–5 segment identifies a relatively small pool (around 4%) of the total number of pontine noradrenergic neurons in the LC and A7 areas (and <2% of A5). Using this vector to express fluorescent markers such as EGFP and immunohistochemistry we have obtained a Golgi-like visualization of the anatomy and projections of the pontospinal noradrenergic neurons. Furthermore, we have been able to show that these pontospinal noradrenergic neurons are activated (express c-fos) during a noxious stimulus applied to the hindpaw, suggesting that these are nociresponsive and are therefore likely to constitute a component of the descending antinociceptive control system (Jones, 1991; Millan, 1997,^2002^; Pertovaara, 2006).

### Organization of the pontospinal noradrenergic system

In agreement with a number of previous studies, we have shown that the dense noradrenergic innervation of the dorsal horn originates from neurons whose cell bodies are located in the pontine A5, LC, and A7 regions (Westlund et al., 1983; Fritschy and Grzanna, 1990; Clark and Proudfit, 1991a,b,^1993^). In total, our viral vector identifies around 150 pontine neurons with terminal projections to the L4–5 dorsal horn. Previous ablation studies using intrathecal administration of anti-DBH-saporin showed loss of the majority of neurons in LC (Jasmin et al., 2003a). However, those authors concluded that their toxin had spread in the CSF to reach higher centers such as the cortex, thus lesioning a large proportion of the NA neurons. The AVV-PRS-EGFP by contrast requires intraparenchymal administration to target the pontospinal neurons so there is no spread in the CSF to higher centers.

The majority of our retrogradely labeled neurons were located in LC (80%), with the remainder in A5 (12%) and A7 (8%). This suggests that the predominant source of the neurons projecting to the dorsal horn is the LC. However, when considered as a proportion of the total number of noradrenergic neurons in each nucleus, the distribution becomes more even across the pontine nuclei LC (4.4%) and A7 (4%) and with relatively fewer in A5 (1.4%). These proportions suggest that the LC and A7 nuclei may have topographical organizations with similar weightings for the hindlimb spinal segments.

Our adenoviral vector strategy identified comparatively fewer pontospinal noradrenergic neurons than chemical retrograde tracers (FG/CTb injected into the same region of the spinal cord) with ≈50–70% the number of LC neurons (115 ± 10 [AAV] vs. 244 ± 8 [FG] or 161 ± 51 [CTb]) and relatively smaller proportions of neurons in the A5 and A7 areas. It should be noted, however, that both FG and CTb spread considerably further within the cord parenchyma than the AVV to extend several segments rostrocaudally and throughout the full dorsoventral extent of the cord, thus they are likely to have labeled the pool of NA neurons projecting to the lumbar enlargement. In particular, the intermediolateral cell column, which terminates at L2, would have been included in the field of the conventional tracer injection but not in our AVV injections. This may account for the differences in the relative distribution of retrogradely labeled neurons across the NA nuclei, particularly in the case of the A5 region (and in the rostral ventrolateral medulla,) which have prominent projections to the sympathetic preganglionic neurons in the intermediolateral cell column (Loewy et al., 1979). In addition, it seems likely that our viral vector is only taken up by axon terminal fields (see below and Ridoux et al., 1994; Liu et al., 1997b), unlike FG or CTb, which are known to be taken up by axons of passage and therefore are likely to label noradrenergic neurons with projections traveling to more caudal spinal targets. By contrast, retrograde labeling with fluorescent latex microspheres (whose distribution was more limited in the spinal cord) identified comparable numbers of neurons as the AVV in the pontine A5, LC, and A7 areas with a similar proportional distribution across these nuclei. Hence, it would appear that dorsal horn injections of our AVV produces focal transduction of the NA projection neurons that is of comparable efficacy to conventional tracers and, based on these findings, we have no reason to believe that our AVV is selectively targeting a specific subgroup of the pontospinal neurons. Thus, in agreement with previous studies (Guyenet, 1980; Loughlin et al., 1986), we find that these pontospinal projections originate from a limited subset of the total population of NA neurons.

The topography of the pontospinal projection was characterized using AVVs expressing either EGFP or mRFP. This showed that the noradrenergic projection to the cord from the pons is bilateral, with an ipsilateral predominance (62%) in agreement with previous functional studies showing bilateral analgesic effects from unilateral stimulation of LC (Jones and Gebhart, 1986b). A proportion of LC neurons were found to have bilateral projection fields (at least 4% of the pontospinal neurons). We found relatively little overlap in the neurons projecting to the cervical and lumbar spinal dorsal horn (L4–5 and C5–6, 1% colocalization). By contrast, co-injection of the vectors (or fluorescent microspheres) in the same sites in the lumbar spine produced ≈60% colocalization. This indicates that there is a rostrocaudal topographical organization of the pontospinal noradrenergic projection, with little overlap in the populations of neurons innervating spinal territories receiving sensory inputs from the fore- and hindlimbs. In addition, the fact that we observed little double labeling when making combined AVV injections to lumbar and cervical cord suggests that the AVV is only retrogradely transported from the terminal axonal field rather than by axons of passage (unlike conventional retrograde tracers). This makes the AVV a particularly selective tool for retrograde targeting.

We noted that the EGFP expression allowed the axons of the NA neurons to be identified and this could be enhanced by IHC to give a Golgi-like visualization of distal projections (note there was no increase in the numbers of GFP-positive labeled noradrenergic cell bodies after this immuno-enhancement). A similar ability to define the axonal processes of medullary NA neurons of the (C1 group) has been reported using direct injection of a lentiviral vector employing the PRS promoter to drive EGFP expression (Card et al., 2006). We found that the pontospinal neurons showed the expected pattern of axon terminal fields in the spinal cord. When comparing the density of the EGFP-containing fibers with those revealed by DBH IHC it was apparent that even in territories with relatively dense EGFP-containing projections (such as the superficial dorsal horn) there was a greater density of DBH-containing fibers. Given the distance of these axonal terminals from the NA somata, where the EGFP synthesis occurs, this disparity is likely to be a consequence of a concentration threshold effect for the visualization of EGFP-containing axons by immunocytochemistry.

Interestingly, axonal projections from the pontospinal NA neurons were also seen in other regions of the neuroaxis, such as the inferior olive, periaqueductal gray, cerebellum, thalamus, and some regions of the cortex (insular, cingulate, and piriform). Using the injection of fluorescent microspheres to the thalamic VPL we were able to retrogradely label neurons in the LC (as has been previously shown; Simpson et al., 1997; Voisin et al., 2005) and the presence of double labeling confirmed that some pontospinal LC neurons also project to the thalamus. We found no evidence of double labeling of pontospinal A5 or A7 neurons from the VPL. The distribution pattern of EGFP-containing fibers from the pontospinal neurons was specific, as there was no projection seen to some regions that have a dense noradrenergic innervation (DBH IHC), such as the hippocampus, caudate putamen, nucleus accumbens, and entorhinal cortex. This restricted distribution is in agreement with previous findings showing a topographical organization of the neurons in LC (Loughlin et al., 1986). The distributed nature of the NA system and the lack of conventional synaptic terminals (Descarries et al., 1977) have led to the inference that these neurons exert a diffuse global effect whose synchronized activation is important for generalized phenomena such as arousal (see Dismukes, 1977). However, the anatomical organization of the NA neurons projecting to the spinal cord suggests that they exert influence over a limited range of neural territories that share common functional specificity, e.g., pain matrix (insular and cingulate cortices, thalamus, periaqueductal gray, parabrachial nucleus, and spinal dorsal horn) or motor control (cerebellum, thalamus, and inferior olive).

### Methodological considerations

In agreement with previous studies, we have shown AVV to be capable of retrograde transduction of neurons (Akli et al., 1993; Ridoux et al., 1994; Liu et al., 1997b; Lonergan et al., 2005; Tomioka and Rockland, 2006). Following spinal injection of the AVV, EGFP-positive pontine neurons were first seen after 2–3 days and labeling was maximal at 7 days. Efficient retrograde labeling required higher titers of spinal AVV (>10^10^ TU/mL). However, above this level a significant fall-off was seen in the numbers of transfected pontine neurons, perhaps because local spinal reactions around the injection site damaged axon terminals before transduction could be initiated. Such an inflammatory effect has been previously reported following spinal injections of high concentrations of AVV (Liu et al., 1997b).

The cell-specific promoter PRS successfully restricted expression of EGFP to pontine noradrenergic neurons as shown by DBH IHC. Previous spinal administration of AVV with the cytomegalovirus promoter has shown labeling of brainstem neurons in the red nucleus, vestibular nuclei, within the pontine reticular formation, and the locus coeruleus (Liu et al., 1997b). By contrast, we only saw expression in the A5, LC, and A7 cell groups with the PRS promoter. There is evidence that the PRS promoter is active in noncatecholaminergic neurons that express the Phox2 transcription factor, such as cholinergic autonomic neurons in the brainstem in areas like the dorsal vagal motor nucleus (Tiveron et al., 1996; Brunet and Pattyn, 2002; Lonergan et al., 2005). Following spinal administration of AVV-PRS-EGFP, we saw no expression in these brainstem cholinergic groups, as they do not project to the spinal cord. Furthermore, the spinal motoneurons and sympathetic preganglionic neurons, which are cholinergic, do not express the Phox2 transcription factor (Tiveron et al., 1996) and we saw no expression of EGFP in these cell groups following viral injection.

It is worth noting that following spinal injection of AVV the rats showed a prompt recovery of motor and sensory function, such that their rotarod test was normal, as were their responses to thermal and mechanical sensory stimuli applied to the hindpaw (unpublished observation). Furthermore, rats transfected with AVV-PRS-EGFP showed typical biphasic nociceptive behavioral responses to formalin testing. Thus, our spinal dorsal horn injections of AVV were not associated with discernable gross motor or sensory deficits. Also, within the brainstem the retrograde transduction with AVV of the NA neurons appeared to be well tolerated. These neurons are capable of producing increased levels of c-fos following noxious stimulation. We saw no change in the numbers of labeled pontine NA neurons at timepoints of up to 1 month, indicating that there was no cell death as a consequence of AVV transduction and that the gene product continues to be transcribed for this time period. This is in contrast to the experience with high titers of AVVs when used by direct injection into the target cell groups where gene expression appears short-lived, in part because of local immune activation (Thomas et al., 2001; Kugler et al., 2003). We have also made electrophysiological recordings from transfected LC cells in both acute slices and also in slice cultures of the pons (unpublished observation) and the neurons appear healthy with firing patterns and intrinsic conductances similar to those previously reported (Williams and Marshall, 1987).

The use of replication deficient viral vectors offers a number of useful features compared to conventional neuroanatomical tracing techniques. Through the use of cell-specific promoters it is possible to express the gene of interest selectively in a particular phenotype of cell, e.g., neuronal or glial (Lonergan et al., 2005; Card et al., 2006). Furthermore, there are a number of different fluorophores (e.g., EGFP or mRFP) or histochemical markers (i.e., beta-galactosidase) that can be expressed and used to label different cell populations using the same type of viral vector and promoter system. They also have the potential to allow anatomically targeted functional manipulations through the expression of genes that alter neuronal function (Johns et al., 1999). In the exploration of the noradrenergic system, this could simply be an extension of the lesion experiments, which have used either metabolic toxins such as DSP4 (Zhong et al., 1985) or immuno-toxins such as anti-DBH-saporin (Zhong et al., 1985; Martin et al., 1999; Jasmin et al., 2003a). However, the major advantage of the viral vectors is that they can allow manipulations to up- or downregulate specific neuronal functions without ablating the neurons of interest (e.g., Lechner et al., 2002; Duale et al., 2007).

### Functional activation

To examine the role of the pontospinal NA neurons in nociception, we employed the formalin test (Dubuisson and Dennis, 1977) as a model of a persistent acute pain, as it has previously been shown that the endogenous NA analgesic system is actively involved in attenuating the response to this stimulus (Sugimoto et al., 1986; Liu et al., 1997a; Omote et al., 1998). It has also been shown that LC NA neurons show increased expression of c-fos in response to noxious stimulation such as hindpaw injections of formalin (Baulmann et al., 2000) or carrageenan (Tsuruoka et al., 2003) and also following tooth pulp stimulation (Voisin et al., 2005). Tachykinin receptor antagonists attenuated both the behavioral and increased LC c-fos responses to formalin, suggesting that the LC neurons are nociresponsive (Baulmann et al., 2000). Indeed, this increase in LC c-fos expression has been suggested to be a marker of the activation of the descending antinociceptive control system (Tsuruoka et al., 2003). Our study demonstrates that the pontine-lumbospinal NA neurons express c-fos suggesting that they are activated during the formalin test. This finding is similar to that reported by Voisin et al. (2005), who showed c-fos expression in LC neurons projecting to the somatosensory (VPL) thalamus after electrical tooth pulp stimulation. It is interesting to note that some of our pontospinal neurons were shown to also project to the somatosensory thalamus and thus may be capable of exerting a coordinated, multilevel regulatory influence on noxious transmission. We have also shown that similar proportions of pontospinal neurons are activated across all three of the noradrenergic cell groups (A5, LC, and A7). This suggests that they all make a contribution to the response, but it remains to be determined whether they are exerting similar effects, acting cooperatively via different mechanisms or whether they produce antagonistic effects.

In summary, we have demonstrated the use of an AVV to effectively and selectively target the neurons of the noradrenergic pontospinal projection to the lumbar dorsal horn. We show that these neurons form a subgroup (<5%) of the total pool of pontine NA neurons and that their spinal projection has a distinct rostrocaudal topology. We show that at least some of the LC pontospinal neurons project to other regions in the brainstem and higher centers, which are recognized as part of the pain matrix providing the substrate for a multilevel response to noxious inputs. Finally, we show that these neurons are activated in response to a persistent noxious stimulus indicating that they are likely to be a component of the endogenous analgesic system. There is good evidence that this NA system is involved in the control of pain and nociceptive behaviors (Millan, 2002; Pertovaara, 2006), pharmacological manipulation of this system is useful in the treatment of both acute and chronic pain (Eisenach et al., 1995; Sindrup and Jensen, 1999) and there is evidence of dysregulation this system in models of neuropathic pain (Ma and Eisenach, 2003). Using this type of viral vector targeting of the pontospinal neurons we may in principle be able to express gene products (e.g., ion channels) to manipulate the activity of noradrenergic neurons to suppress pain signals originating from a particular spinal level, such as those associated with certain severe neuropathic pains such as postherpetic neuralgia or following spinal cord injury.
