# Supplementary figures and images for: Retrograde adenoviral vector targeting of nociresponsive pontospinal noradrenergic neurons in the rat in vivo
Source: J Comp Neurol. 2009 Jan 10;512(2):141–57. doi: 10.1002/cne.21879 (PMC2659361; doi:10.1002/cne.21879)

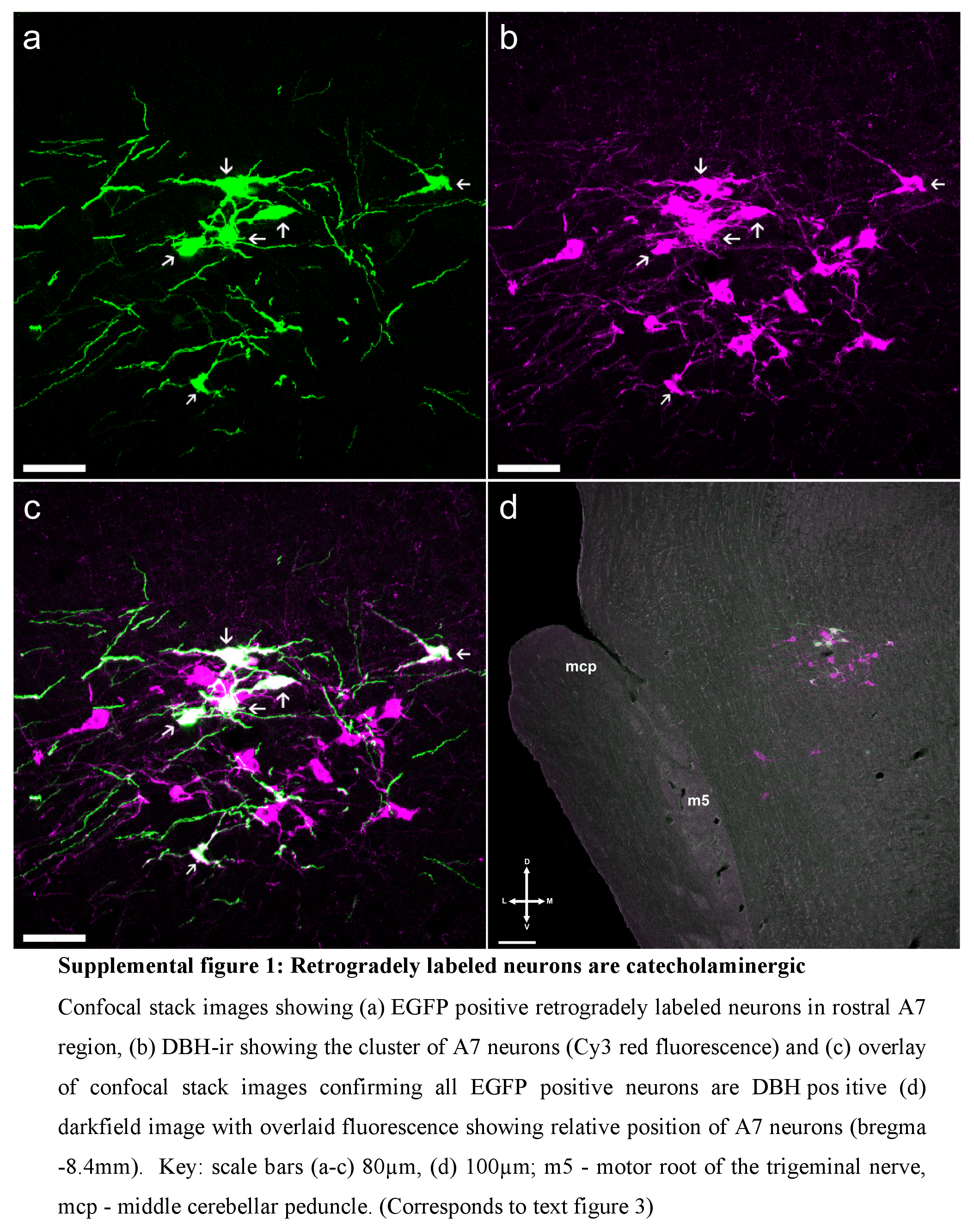

Supplement: Supplementary file 1 [file cne0512-0141-SD1.tif]

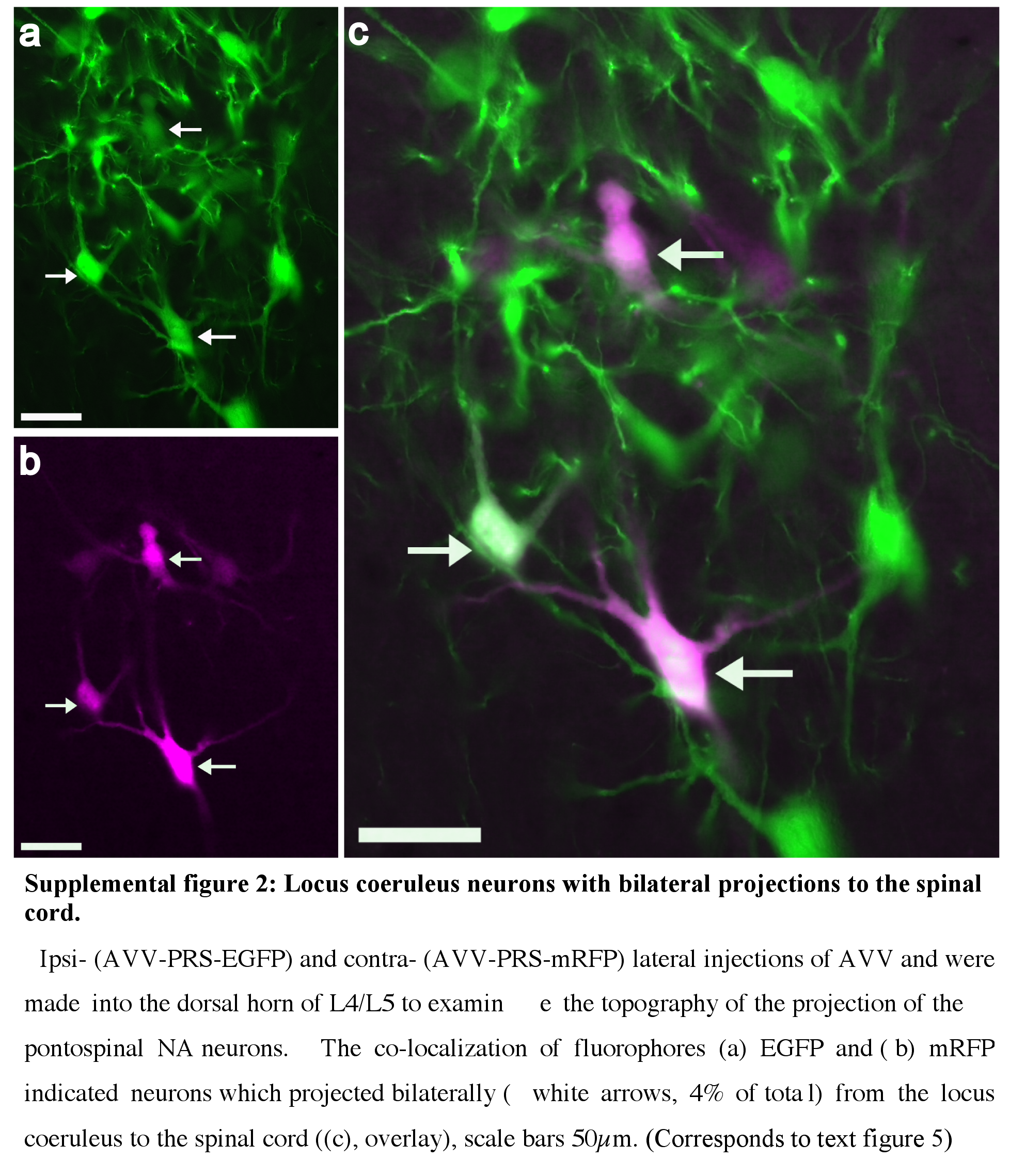

Supplement: Supplementary file 2 [file cne0512-0141-SD2.tif]

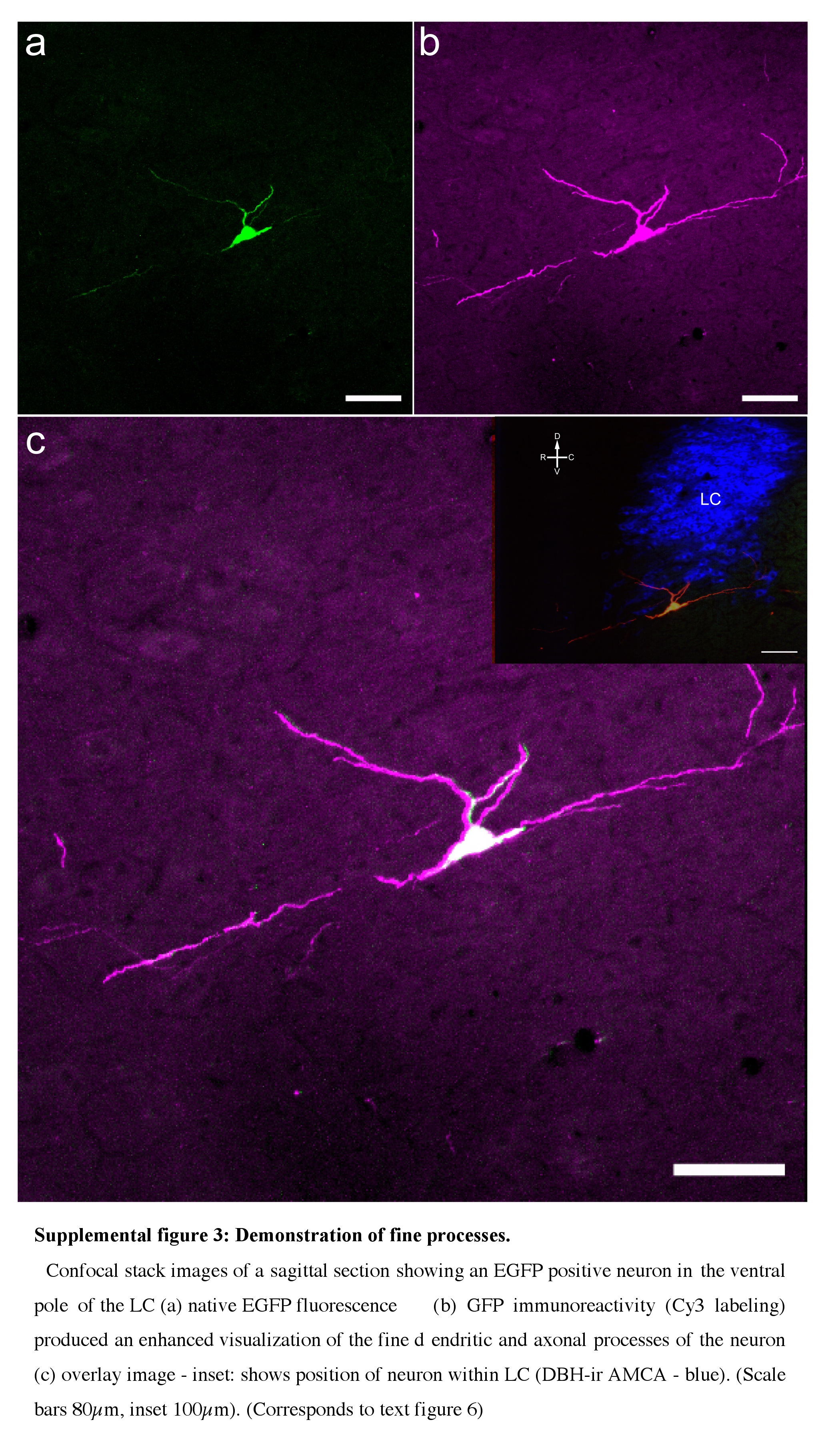

Supplement: Supplementary file 3 [file cne0512-0141-SD3.tif]

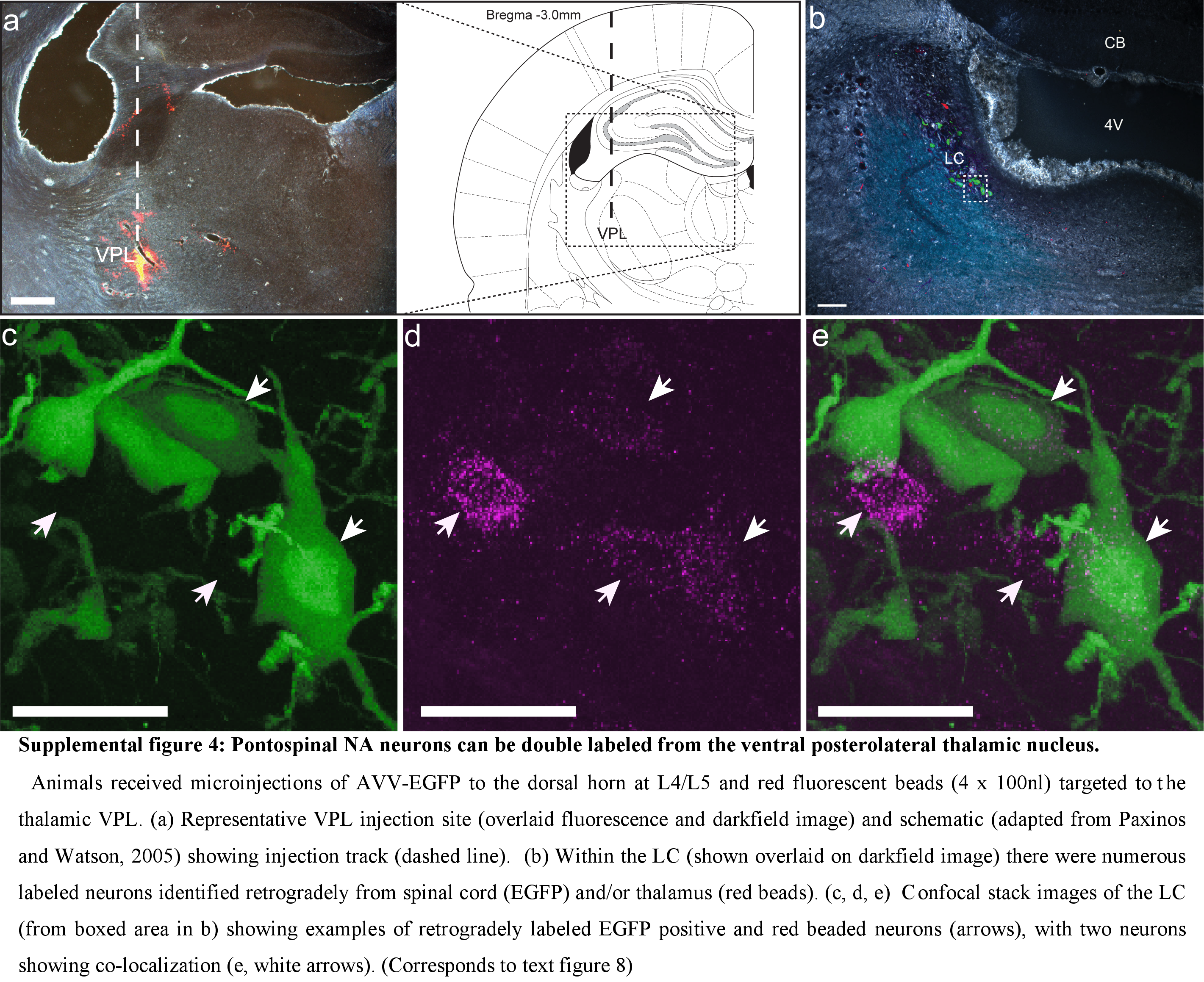

Supplement: Supplementary file 4 [file cne0512-0141-SD4.tif]
